# Recent Developments on Bioinspired Cellulose Containing Polymer Nanocomposite Cation and Anion Exchange Membranes for Fuel Cells (PEMFC and AFC)

**DOI:** 10.3390/polym14235248

**Published:** 2022-12-01

**Authors:** Sadhasivam Thangarasu, Tae-Hwan Oh

**Affiliations:** School of Chemical Engineering, Yeungnam University, Gyeongsan 38541, Republic of Korea

**Keywords:** proton exchange membrane fuel cell, alkaline fuel cell, ion exchange membrane, biopolymer, cellulose membrane, cation exchange membrane, anion exchange membrane

## Abstract

Hydrogen fuel cell (FC) technologies are being worked on as a possible replacement for fossil fuels because they produce a lot of energy and do not pollute the air. In FC, ion-exchange membranes (IEMs) are the vital components for ion transport between two porous electrodes. However, the high production cost of commercialized membranes limits their benefits. Various research has focused on cellulose-based membranes such as IEM with high proton conductivity, and mechanical, chemical, and thermal stabilities to replace the high cost of synthetic polymer materials. In this review, we focus on and explain the recent progress (from 2018 to 2022) of cellulose-containing hybrid membranes as cation exchange membranes (CEM) and anion exchange membranes (AEM) for proton exchange membrane fuel cells (PEMFC) and alkaline fuel cells (AFC). In this account, we focused primarily on the effect of cellulose materials in various membranes on the functional properties of various polymer membranes. The development of hybrid membranes with cellulose for PEMFC and AFC has been classified based on the combination of other polymers and materials. For PEMFC, the sections are associated with cellulose with Nafion, polyaryletherketone, various polymeric materials, ionic liquid, inorganic fillers, and natural materials. Moreover, the cellulose-containing AEM for AFC has been summarized in detail. Furthermore, this review explains the significance of cellulose and cellulose derivative-modified membranes during fuel cell performance. Notably, this review shows the vital information needed to improve the ion exchange membrane in PEMFC and AFC technologies.

## 1. Introduction

The search for alternative energy has recently been a concern worldwide due to fossil fuel diminution and environmental complications [1,2,3,4]. Over the past few decades, fossil fuels have been significantly extracted from the earth’s crust to accomplish the energy needs of humans and others. However, the energy derived from fossil fuels cannot fulfill future energy needs because of the highest energy utilization by rapid population growth worldwide [5,6]. Additionally, environmental-related worries are also associated with fossil fuel-related energy. When fossil fuels are converted into energy, they release a considerable amount of CO_2_ and many other greenhouse gases as a byproduct, which significantly spoil the good environment [7,8]. Based on this apprehension, researchers worldwide focused on various ideas and developments to derive energy in a clean and green form from renewable energy sources like solar, wind, geo, hydro, and bio energies [9,10,11]. In this connection, “hydrogen” as a green energy fuel can be produced using energy derived from renewable sources [12,13].

Hydrogen is considered a promising alternative fuel because of its energy. The chemical energy in hydrogen is nearly three times greater than that of typical fossil fuels. As an example, the chemical energy of hydrogen is 141.865 MJ/kg, whereas gasoline and diesel get chemical energy of 45.58 and 45.4 MJ/kg, respectively [13,14]. “Hydrogen Energy” is majorly classified into three essential sectors, hydrogen production, hydrogen storage, and hydrogen distribution [15,16]. Using numerous techniques, hydrogen can be produced from various sources, such as natural gas, oil, coal, and electrolysis [17,18]. “Hydrogen production” via the water splitting process (photocatalyst or electrocatalyst) is an efficient option, where high-purity hydrogen can be obtained without any harmful byproducts [19,20]. Hydrogen gas is one of the lightest material (0.08988 g/L) in the universe [21,22]. Hydrogen can be stored (“hydrogen storage”) in different forms, such as gaseous, liquid, and solid. In the gaseous state, liquid state, and solid state, the hydrogen is stored as pressurized gas, cryogenic liquid, and bonded (physical/chemical) with solid materials, respectively [23,24,25]. The solid state of hydrogen storage can be further classified into broad categories like physical adsorption (e.g., large surface area materials) and chemical absorption (intermetallic hydrides, elemental hydrides, complex hydride and chemical hydrides) [26,27,28]. To store a higher amount of hydrogen in a less volumetric and feasible way, solid-state hydrogen storage has been considered a promising way. In recent periods, the commercialization of fuel cell vehicles for on-road has provided most specific interests [29,30]. The “hydrogen distribution” via fuel cell is the ultimate use of hydrogen energy for practical application [29,31,32,33].

**Figure 1:**
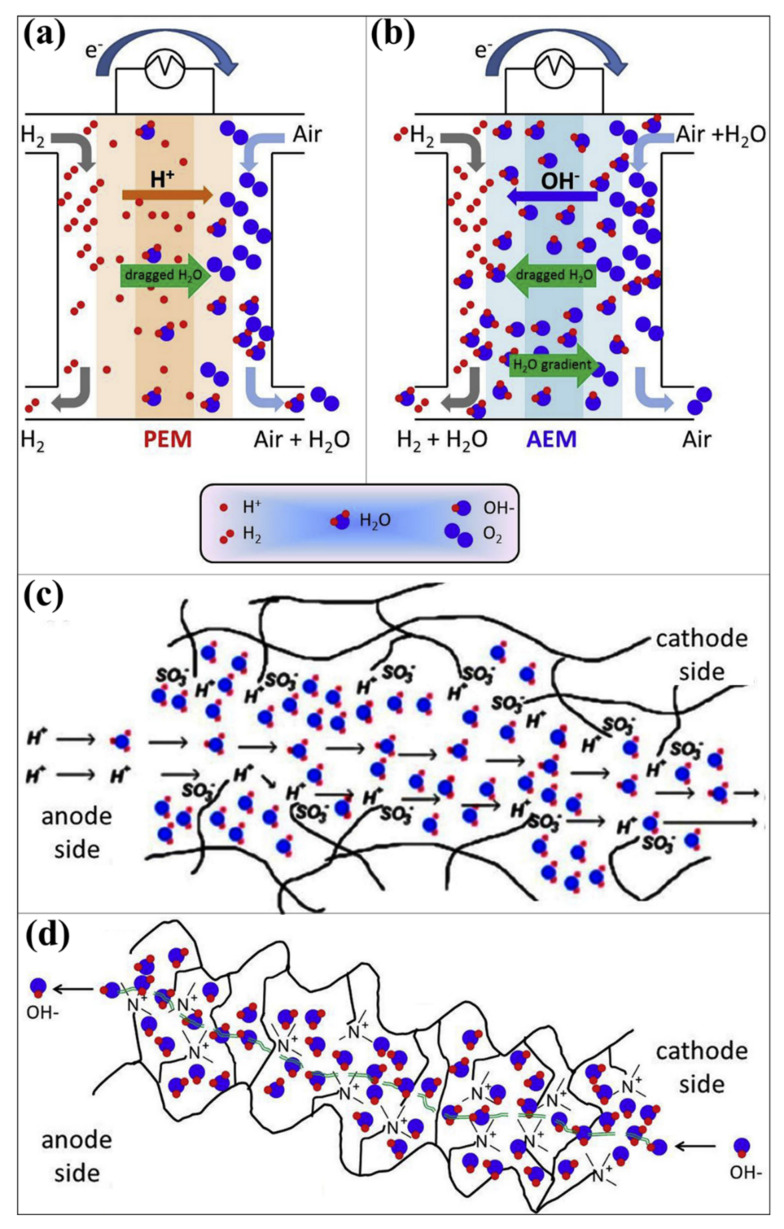
Schematic representation of (**a**) PEMFC and (**b**) AFC working mechanism. (**d**) Anion transport mechanism in the AEM. Reprinted with permission from Ref. [44]. Copyright © 2017 The Author. Published by Elsevier B.V. (License Number: 5413020972545). (**c**) Cation transport mechanism in the CEM. Reprinted with permission from Ref. [50]. Copyright © 2021 Science Press and Dalian Institute of Chemical Physics, Chinese Academy of Sciences. Published by ELSEVIER B.V. and Science Press. (License Number: 5430181378139).

There are numerous kinds of fuel cell technologies have been established. It has been further classified by the fuels, electrolytes, and operating conditions such as proton exchange membrane (or) polymer electrolyte membrane fuel cell (PEMFC), alkaline fuel cell (AFC) or Bacon fuel cell, phosphoric acid fuel cell (PAFC), molten carbonate fuel cell, solid oxide fuel cell, direct formic acid fuel cell, enzymatic fuel cell, direct borohydride fuel cell, direct carbon fuel cell, microbial fuel cell, direct ethylene glycol fuel cell, direct ethanol fuel cell, direct methanol fuel cell, and alkaline direct methanol fuel cell [34,35,36,37,38,39,40]. The “hydrogen gas” is commonly used in the following “fuel cells”: PEMFC, AFC, PAFC, molten carbonate fuel cells, and solid oxide fuel cells. Among these, most consideration relates to the PEMFC and AFC because of their operating temperature, which can correspond to the room temperature [41,42]. Moreover, hydrogen was applied to PEMFC and AFC as fuel in the anode side, and the reaction happened without any other harmful gases and byproducts [43]. The operating principle and reaction process of PEMFC and AFC has been schematically illustrated in Figure 1a and Figure 1b, respectively [44,45]. In both cases, the membrane is the centerpiece of the unit cells and plays the major role in completing the unit cell operation [46,47]. In PEMFC, the cation exchange membranes (CEM) play a significant role in transporting the proton (H^+^) from the anode side to the cathode side (Figure 1c) [48,49,50]. As shown in Figure 1c, the CEM transports the proton in two possible ways, the Grotthuss mechanism and the vehicle mechanism. In the vehicle mechanism, the protons are diffused through a hydrated membrane using water molecules, where solvated protons form H_3_O^+^ for diffusion. In the Grotthuss mechanism, proton transportation occurs via proton hopping by creating hydrogen bonds in the hydrolyzed ionic site of the membrane [48,49,50]. In AFC, the anion exchange membranes (AEM) play a major role in transporting the anion (OH^−^) from the cathode side to the anode side (Figure 1d) [44,51]. The presence of water molecules in the AEM influences the OH^−^ ion transportation [51]. In both cases, the membrane choice is highly important for obtaining excellent fuel cell performances [52,53,54,55,56]. Commonly, the following requirements must be considered for efficient membrane candidates [56,57,58,59].

- Low production cost
- Abundance in nature
- Facile manufacturing process without complicated steps
- Higher ion exchange capacity
- Excellent ion transport behavior
- Reasonable water uptake behavior
- Efficient mechanical stability
- Good thermal properties
- Higher chemical and electrochemical stability
- Insulating properties (to avoid the short circuit between cathode and anode)
- Lowest cross-over of oxygen and hydrogen
- Environmental friendly

Commercial membranes can meet most of the above-mentioned requirements to serve as an efficient AEM or CEM for fuel cell systems [60]. However, the high cost of commercial membranes restricts their benefits [61,62]. For example, the cost information of some commercial membranes has been provided as follows: (i) Nafion™ 117 membrane: $657.00 (Product Number: 274674; Long side chain PFSA membrane; L × W × T = 8 in. × 10 in. × 0.007 in.; https://www.sigmaaldrich.com/US/en/product/aldrich/274674; accessed on 22 November 2022); (ii) Aquivion^®^ E98-05S: $165.00 (Product Code: 72,700,008; Short side chain PFSA membrane; L × W × T = 30 cm × 30 cm × 50 µm; https://www.fuelcellstore.com/fuel-cell-components/membranes/cation-exchange-membrane/solvay-aquivion-e98-05s 72700006; accessed on 22 November 2022); (iii) Fumasep FKS-PET-130: $86.00 (Product Code: 5,041,616; PET reinforced CEM; L × W × T = 20 cm × 30 cm × 110–130 µm; https://www.fuelcellstore.com/fuel-cell-components/membranes/cation-exchange-membrane/fumasep-fks-pet-130; accessed on 22 November 2022). Moreover, the construction of unit cell prices increases accordingly, limiting its practical application. Thus, numerous kinds of CEM and AEM membrane materials have been developed to decrease the cost and enhance the performance of PEMFC and AFC systems [63,64,65,66,67]. In this context, over the past few decades, considerable attention has been paid to biomaterial-based membranes for fuel cell systems where the biomaterials are very cheap, more easily available, and have efficient intrinsic properties [68,69,70]. In this connection, the higher hydroxyl functional group-containing cellulose (cellulose nano/microcrystal, cellulose nanofibers, and bacterial cellulose) and its derivatives have been studied as a membrane candidate for fuel cell applications. Therefore, in this review, we focused on and explained the recent developments (from 2018 to 2022) of cellulose-containing membranes for PEMFC and AFC. Initially, we explained the fundamental properties of cellulose structure-based materials. We concentrated on the effect of cellulose materials on the functional properties of various membranes. Hybrid membranes made with cellulose have been put into different groups based on how they combine with other polymers and materials. For PEMFC, the sections are associated with cellulose with Nafion, polyaryletherketone, various polymeric materials, ionic liquid, inorganic fillers, and natural materials. Moreover, the cellulose-containing AEM for AFC has been summarized in detail.

## 2. Developments of Different Cellulose Materials

**Figure 2:**
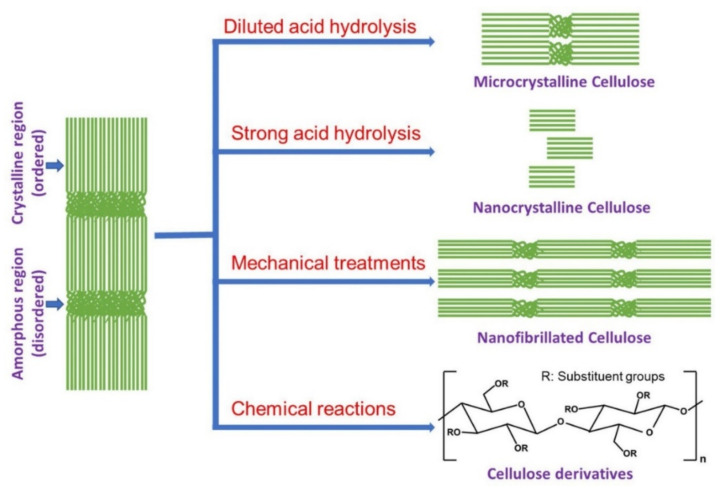
The development processes for several forms of cellulose. Reprinted with permission from Ref. [74]. Copyright © 2020 Elsevier Ltd. (License Number: 5413021363165).

Cellulose (C_6_H_10_O_5_)_n_, the most abundant natural polysaccharide, consists of thousands of D-glucose units linked by β(1→4) glycosidic linkages. Apart from plants (a primary source), different microorganisms, namely bacteria and fungi, also generate cellulose. Generally, cellulose architecture is composed of crystalline and amorphous domains depending on the polymerization degree and chain length. Based on their source and extraction method, up to 40 to 70% of cellulose crystallinity has been varied. Moreover, the physical, chemical, and enzymatic treatments do not influence the crystalline domains, whereas low-density amorphous domains tend to react with other molecular groups. Depending on the molecular alignment, van der Waals force, intra-intermolecular synergy, extraction, and treatment, different types of polymorphs such as cellulose I, II, III_1_, III_2_, IV_1,_ and IV_11_ have been found. With the aid of chemical or thermal modifications, the polymorphs can be transferred from one form into another [71]. The strong hydrogen bonds in cellulose are responsible for its stability and soluble in only particular solvents [72]. Cellulose has been mentioned in different forms based on its size ranging from larger to smaller units (eukaryotic kingdom) and tiny to smaller units (prokaryotic kingdom) [73]. Based on their structure, morphology, and molecular structures, cellulose is classified as microcrystalline cellulose (MCC), nanocrystalline cellulose (CNC), cellulose nanofibers (CNF), also called nanofibrillated cellulose (CNF), bacterial nanocellulose, and cellulose derivatives (e.g., cellulose acetate) (Figure 2) [74].

Microcrystalline cellulose is chemically derived from lignocellulosic biomass with more crystalline parts [75]. MCC is developed from different sources with varied particle sizes and crystalline natures (5% to 80%). The MCC’s physical, chemical, and stability characteristics depend on acid concentration, reaction time, temperature, source raw materials, acid type, and solid loading [75,76,77]. Nanocrystalline cellulose, also called cellulose nanocrystals (CNC), are needle/whisker-like nanoparticles with a size of less than 100 nm and an enhanced crystalline nature [78]. Generally, the acid hydrolysis (using sulphuric acid, 64 wt.%) of cellulose microfibrils with specific time and temperature results in the elimination of amorphous region results in CNC with nanoscale dimensions [79]. Apart from this, the phosphotungstic acid in an acetic acid medium eliminates the cotton cellulose for preparing CNC [80]. During cellulose acid hydrolysis, the crystalline part of the cellulose is isolated, and sulfate groups are grafted onto a CNC surface that aids the cellulose stability because of the electrostatic repulsive forces of negatively charged surface sulfate groups [81]. Through this acid treatment, the rod-like/whiskers or needle-like CNC is largely produced, and spherical CNC is synthesized by fiber pre-swelling along with acid hydrolysis [82]. The substantial dispersion of CNC obtained from sulfuric acid and phosphoric acid, while poorly dispersible, was observed in CNC from hydrochloric acid hydrolysis [83]. Cellulose nanofibers (nanofibrillated cellulose or bacterial nanocellulose) are considered based on diameter, which is nano size, and though its length has been found to be a few micrometers, it falls under the nanocellulose category [74]. The CNF has been isolated by separating the microfibril and overwhelming the complexity in inter-fibrils hydrogen bonding and physical interactions in cellulose bundles [84]. To obtain CNF of a particular size, the treatment condition parameters are modified. The development of CNF reduces agricultural waste disposal and upgrades it into high-performance materials [78]. The derivative of cellulose, namely cellulose acetate, is produced via homogenous or heterogeneous functionalization. In heterogeneous method, the more crystalline and low-biodegradable cellulose acetate has fabricated by acetic anhydride and acetic acid with sulphuric acid or pyridine as catalyst that acetylating the cellulose hydroxyl groups [85,86]. During homogeneous functionalization, the cellulose acetate is produced with a feasible degree of substitution with controlled reaction time, temperature, and acetylating agent without catalysts [87]. Nowadays cellulose containing materials are effectively considered for different kinds of applications such as biomedical applications, bioremediation, and electrochemical energy storage/conversion devices because of its abundance, cost-effectiveness, hydrophilic nature, efficient mechanical and thermal properties, renewability, biocompatibility, and intrinsic properties [88,89,90,91,92,93]. In this way, cellulose and cellulose derivatives gained more attention as ion exchange membranes for different kinds of fuel cells.

## 3. Cellulose Containing Cation Exchange Membrane for PEMFC

To replace the high cost of commercial membranes and further enhance PEMFC performance, various kinds of membranes have been developed through different concepts, such as modification of organic polymers, organic-inorganic hybrid polymer membranes, blend membranes, cross-linked membranes, etc. [94,95,96,97]. In this connection, incorporated cellulose and cellulose derivatives and modified membranes were developed and demonstrated their properties for the PEM fuel cell applications.

### 3.1. Modification of Cellulose

Recently, the modification of cellulose and its derivatives has been of great interest in various energy applications and fuel cells [98,99,100,101,102,103,104,105]. To develop the carboxylated CNF, the cellulose pulp was first oxidized by 2,2,6,6-tetramethylpiperidine-1-oxyl (TEMPO), and then the oxidized pulp was mechanically fibrillated [106]. In situ measurements have been made to evaluate the effectiveness of thin carboxylated cellulose nanofiber-based (CNF) membranes as PEM. This is evaluated depending on the fuel cell’s relative humidity (RH 55 to 95%), membrane thickness, counter ion (H^+^ or Na^+^), and CNF surface charge density (600 and 1550 µmol g^−1^). Water channels only begin to emerge when the relative humidity (RH) is over 75%, as determined by the Small Angle X-ray Scattering technique. It has been determined that the counter ions (H^+^ or Na^+^) and membrane surface charge influence how much water is absorbed. The obtained proton conductivity is greater than 1 mS cm^−1^ at 30 °C between 65 and 95% RH, which is made possible by CNF’s homogeneity and strong affinity for water. In fact, compared to Nafion 212, it contains more than 100 times as many water molecules per proton conducting site. Because CNF membranes have better mechanical qualities than Nafion, they may be made thinner without sacrificing their ability to act as a gas barrier. This might reduce internal cell resistance. Because of the minimal hydrogen crossover, the CNF membranes demonstrate greater open circuit voltages comparable to those of Nafion [106].

Sharma et al. developed nitro-oxidized carboxycellulose nanofibers (NOCNFs) from jute as a PEM [107]. NOCNFs were produced with 2 implementations, carboxylate and carboxylic acid. Afterwards, they were converted into nano-paper I (carboxylate) and II (carboxylic acid). High-acid -COOH groups were employed to crosslink nanofibers via multiple hydrogen bonding contacts. These phenomena give nano-papers a dense and robust structure in addition to serving as proton donors and charge carriers. At 80 °C (at 100% RH), the greatest conductivity for nanopaper II reached 14.6 mS cm^−1^, whereas nanopaper I attained 10.4 mS cm^−1^. Improved proton transport was made possible by the surfaces of nano-paper II’s more acidic -COOH units. As predicted, fuel cells containing nano-paper II performed well with a high power density (19.1 mW/cm^2^). This is due to nanopaper IIs increased tensile strength, hydrophilicity, dense surface, and conductivity. To create high-temperature membranes, nitro-oxidized nanocellulose paper may be used as a cost-effective, environmentally friendly, and sustainable source [107]. In another method, the sulfonation functional group is introduced in CNF to enhance the ion exchange capacity (IEC) and ionic conductivity (IC) of CNF membranes [108]. Sodium periodate and odium bisulfite was used to oxidize and then sulphonate the CNF. In this study, fuel cells are constructed using CNF “paper” membranes of 30 m thick. The IEC significantly increases to CNF after sulphonation, where the IEC is 0.016 mmol g^−1^ and 0.900 mmol g^−1^ for CNF and sulfonated CNF, respectively. Cellulose nanofibers have been sulfonated to increase through plane conductivity (up to 2 × 10^−3^ S cm^−1^) without sacrificing membrane integrity. Moreover, the determined water uptake (and swelling ratio) of the sulfonated CNF membrane is 26 ± 3% (14 ± 5%), which is comparable to the Nafion membrane 26 ± 2% (16 ± 2%). Compared to Nafion fuel cells of equal thickness, the hydrogen crossover current is normally lower, although the energy capacity is very poor. Under standard measurement circumstances (0.1 MPa; 95% RH; 80 °C; H_2_/air), the resultant paper fuel cell displays high power density (156 mW cm^−2^) and current density (>0.8 A cm^−2^). This efficient performance has ascribed to lower membranes impedance [108].

**Figure 3:**
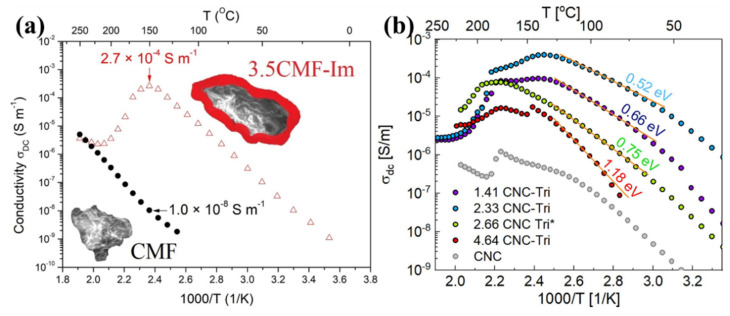
Proton conductivity of (**a**) cellulose microfibers functionalized with imidazole molecules. Reprinted with permission from Ref. [109]. Copyright © 2019 Elsevier B.V. (License Number: 5413030049297). (**b**) CNC doped with 1H−1,2,3 triazole [110].

The modification of a cellulose surface by imidazole functionalities can possibly enhance the proton transport behavior. Jankowska et al. used imidazole compounds to functionalize cellulose microfibers (CMF) [109]. The proton transportation of imidazole containing CMF may be influenced by the possibility of imidazole separating into an imidazole anion and an imidazolium cation. At 150 °C, the highest conductivity of the novel imidazole-cellulose material is 2.7 × 10^−4^ S m^−1^, where the conductivity of pure cellulose is 1 × 10^−8^ S m^−1^ under similar conditions as shown in Figure 3a. Protons are transported through the dissociation process in conjunction with proton exchange with cellulose’s hydroxyl groups and hydrogen bond rearrangement. This improves the class of cellulose composites’ proton conductivity [109]. The same research group further evaluated the performance of the modification of cellulose materials, where various amounts of 1H-1,2,3 triazole (Tri) were doped in nanocrystalline cellulose (CNC) [110]. There was no structural change in the cellulose after CNC functionalization with Tri. The substance takes the shape of a semitransparent polymer film. To understand the proton transport behavior, the proton conduction analysis for various films was carried out from 20 to 250 °C in an anhydrous environment. As represented in Figure 3b, with increasing triazole concentration in CNC, the CNC-Tri conductivity increased, while the conductivity of CNC-Tri is superior to CNC. At 135 °C, the 2.33 CNC-Tri material had the greatest conductivity value of 4 × 10^−4^ S m^−1^. Proton transport throughout the cellulose membrane was encouraged by the active group of 1H-1,2,3 triazole. Proton transportation was regulated by triazole ring reorientation, and conductivity performance is compatible with Grotthuss mechanism. Comparing CNC-Tri to CNC treated by imidazole, it was observed that CNC-Tri had improved thermal characteristics [110].

**Figure 4:**
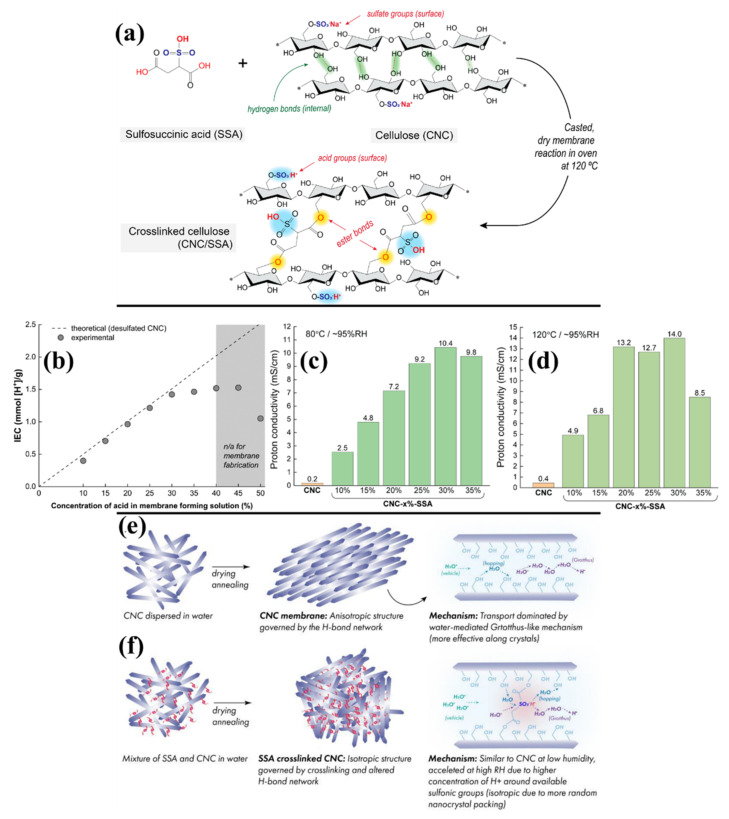
(**a**) An illustration of the cross-linking reaction between sulfosuccinic acid and CNCs, and (**b**) IEC of the CNC−SSA (with different concentrations). Proton conductivity measurement results of different ratios of CNC−SSA membranes (**c**) 80 °C and (**d**) 120 °C. Mechanisms governing membrane development and proton conduction in (**e**) CNC and (**f**) cross-linked CNC [111].

Selyanchyn et al. investigated the impact of sulfosuccinic acid (SSA) cross-linking with CNC with different ratios [111]. SSA contained a core sulfonic acid moiety (-SO_3_^−^H^+^) and two dangling carboxylic acid moieties, which can enhance proton transport behavior after proper crosslinking with CNC. As shown in Figure 4a, the primary reaction route is the development of ester linkages between the CNC hydroxyl group and SSA’s carboxylic group. Among the different concentrations, up to 30% SSA concentration (1.423 mmol/g) with CNC shows higher results (Figure 4b). However, increased SSA levels cause the IEC to saturate and drop below the expected value. Thus, it indicates that the highest quantity of SSA that may be completely incorporated into the membranes as a cross-linker is about 30%. The proton conductivity at 80 °C and 120 °C is represented in Figure 4c and Figure 4d, respectively. A distinct pattern can be seen (at 80 °C and 95% RH), the ionic conductivity sharply rising in direct relation to the SSA concentration up to 30% weight and then somewhat declining at 35% weight. At 80 and 120 °C, the CNC-30%-SSA membrane had maximum ionic conductivity of 10.4 and 14 mS cm^−1^, respectively. The conductivities of SSA cross-linked CNC membranes are much greater than those of CNC membranes, where the CNC membrane attained 0.4 mS/cm proton conductivity at 120 °C. Through-plane conductivity of the CNC-SSA might be improved due to the membrane’s consequent isotropy properties (Figure 4e,f), which is mainly attained by the crosslinking between the CNC and SSA [111].

### 3.2. Cellulose Derivatives with Nafion

In recent periods, the development of cellulose-based blend/composite membranes with different kinds of organic polymers and inorganic additives has been considered to effectively enhance fuel cell performance. According to the blend/composite formation with cellulose materials, the physicochemical properties of the membrane can be further altered. Moreover, various developments have been focused on, together with Nafion, such as blending and composite of various polymeric materials and inorganic fillers to decrease the cost of Nafion-based membranes [112,113]. In this connection, certain developments have already been made to CNC with Nafion [114,115,116]. The impact of different amounts of CNC in the Nafion membrane polymer matrix was investigated [114]. In this case, different lengths of Tunicate and Ramie nanocrystals were considered for developing the CNC. Water absorption capacity, dimensional stability, and Young’s modulus of Nafion membranes were dramatically altered by CNCs, indicating their intense interest in this field. Raising the cellulose concentration (Tunicate or Ramie) in the membrane (0 to 10 wt.%) lowered the in-plane swelling, thickness swelling, and enhanced water absorption. Compared to Nafion and ramie CNC-Nafion (5 wt.%), the 5 wt.% of Tunicate CNC with Nafion was determined to be the efficient mechanical property of the membrane because of reliable Young’s modulus, elongation at break, and tensile strength. Moreover, the 5 wt.% Tunicate CNC with Nafion membrane provides considerable IEC values of 1.07 ± 0.01 meq g^−1^, similar to the Nafion membrane. Interestingly, the proton conductivity of the Nafion membrane further increased after incorporating 5 wt.% Tunicate CNC in the membrane matrix, where the proton conductivity of Nafion and Nafion-5 wt.% Tunicate was 90 mS cm^−1^ and 100 mS cm^−1^, respectively. The membrane properties showed varied aster annealing at temperatures from 100 to 150 °C [114].

**Figure 5:**
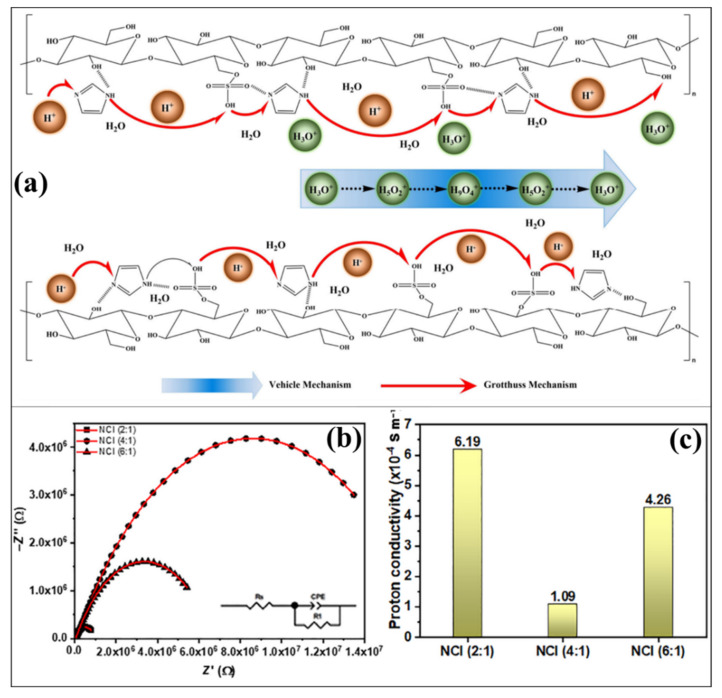
(**a**) Possible proton transport mechanism in sulfonated CNC−Imidazole membrane. (**b**) Nyquist plot and (**c**) proton conductivity of Nafion−CNC−imidazole membranes. Reprinted with permission from Ref. [115]. Copyright © 2021 Society of Chemical Industry (SCI). (License Number: 5413030389680).

A polymer composite structure (CNC/Im) made of doped imidazole (Im) and CNC produced from biomass derived from Vietnamese rice husks was reported by Vu Nang An et al. [115,116], a novel technique for creating Nafion-CNC-imidazole (NCI) composites that uses a relatively facile solution process to combine the Nafion matrix with a CNC/imidazole hybrid composite. Pure CNC has a proton conductivity at 25 °C of 0.19 × 10^−4^ S m^−1^, and doped CNC has a proton conductivity of 1.20 × 10^−4^ S m^−1^ under similar conditions. The vehicular and Grotthuss processes, which make up the main proton transport mechanisms in proton exchange membranes, may explain the CNC/imidazole hybrid composite membrane’s proton conductivity. Because of the hydrophilic channels that the OH and SO_3_^−^ groups on the CNC produced, water molecules remained primarily in the membrane upon synthesis, which helped the vehicular mechanism. As protons move between the nitrogen atoms on the Im and sulfonic acid group in the CNC, the acid-base relationship occurs inside the membrane, which leads to the Grotthuss mechanism (Figure 5a). The advancement of CNC-Im is further tuned by interaction with Nafion ionomer. A different ratio of Nafion and CNC-Im (2:1, 4:1, and 6:1) has been developed to optimize the highest proton conductivity of hybrid membranes. The proton conductivity of Nafion:CNC-Im with different ratios 6:1, 4:1, and 2:1 was 4.26, 1.09, and 6.19 × 10^−4^ S m^−1^, respectively, at 25 °C, as shown in Figure 5b,c. Among different ratios, 2:1 has a value larger than the other samples due to its high amount of CNC (approximately 33%), which may adsorb water molecules more readily [115].

### 3.3. CNC with Polyaryletherketone Family-Based Membranes

**Figure 6:**
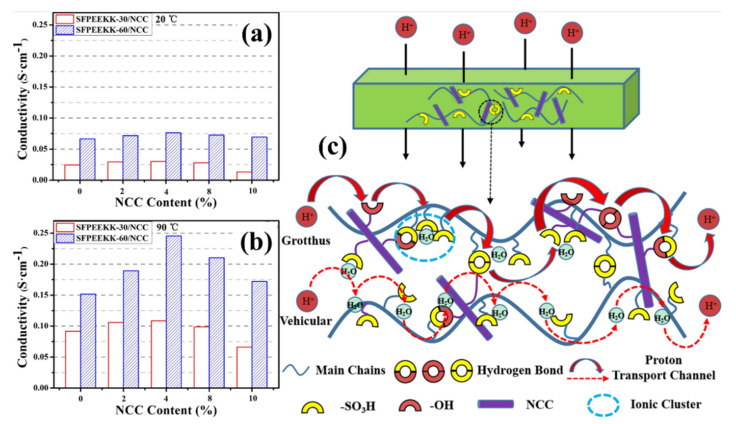
Proton conductivity of different concentrations of CNC and Sulfonated fluorenyl-containing poly(ether ether ketone ketone)s (SFPEEKKs) membranes at different temperatures (**a**) 20 °C and (**b**) 90 °C. (**c**) Illustration showing the CNC−SFPEEKK composites proton transport mechanism. Reprinted with permission from Ref. [117]. Copyright © 2017 Elsevier B.V. (License Number: 5413030592821).

Recently, hydrocarbon polymers have been chosen as one of the efficient alternatives to the high cost of commercial fluorinated polymer membranes. Polyaryletherketone-based polymers have been widely considered promising membrane candidates for fuel cells and flow batteries because of their cost-effectiveness and intrinsic properties. Chuangjiang Ni et al. developed a hybrid composite membrane with sulfonated fluorenyl-containing poly(ether ether ketone ketone) (SFPEEKK) with CNC [117]. Initially, two segments of sulfonated fluorenyl containing poly(ether ether ketone ketone), namely 30% and 60% fluorenyl segments, were in the repeating unit. Based on the fluorenyl segments (30% and 60%) in SFPEEKK, the membranes were termed SFPEEKK-30 and SFPEEKK-60, respectively. Through a solution-casting process, two kinds of SFPEEKK/CNC composite membranes containing different CNC content (0%, 2%, 4%, 8%, and 10%) were created. As mentioned in Table 1 and Figure 6, the CNC with surface modifications was created as the “performance-enhancing” filler in the hybrid membrane. Among the different compositions of SFPEEKK (in both 30 and 60) and CNC, the introduction of 4% CNC provide excellent performance in all kinds of physiochemical characteristics (water uptake, swelling ratio, ion exchange capacity, and proton conductivity). The proton conductivity of SFPEEKK-30 with 4% CNC is superior to the SFPEEKK-30 alone, where the proton conductivity of SFPEEKK-30-4% CNC and SFPEEKK-30 is 0.03 S cm^−1^ at 20 °C (0.109 S cm^−1^ @90 °C) and 0.024 S cm^−1^ at 20 °C (0.092 S cm^−1^ @90 °C). Similarly, the proton conductivity of SFPEEKK-60 with 4% CNC is superior to the SFPEEKK-60 alone, where the proton conductivity of SFPEEKK-60-4% CNC and SFPEEKK-60 is 0.076 S cm^−1^ at 20 °C (0.245 S cm^−1^ @90 °C) and 0.066 S cm^−1^ at 20 °C (0.152 S cm^−1^ @90 °C). As Shown in Figure 6, this result might be explained by the interaction between the SO_3_H/-OH groups on the surface of CNC particles and the SO_3_H groups on the backbone of SFPEEKK, which results in the creation of hydrogen bond networks and proton conduction pathways. The interaction between the SFPEEKK contains SO_3_H groups, and CNC contains -SO_3_H and -OH groups leading to the formation of hydrogen bond networks in the membrane matrix, possibly influencing the enhanced proton conductivity, mechanical properties, and dimensional stability [117]. The same research group further investigated the impact of modified CNC with sulfonated poly(aryletherketone)s with carboxylic acid groups (SPAEK-COOH-x) [118] instead of the SFPEEKK membrane. In SPAEK-COOH-x membranes, the modified CNC is used as a crosslinking agent and performance-enhancing filler. The polymeric membranes incorporating CNC demonstrated greater proton conductivity and enhanced mechanical characteristics. The covalently cross-linked composites membrane had significantly superior mechanical characteristics after additional crosslinking while maintaining its thermal properties and ion conductivity. Among the various concepts, the CNC cross-linked with SPAEK-COOH-10 provides the required thermal properties, mechanical stability, and proton conductivity. Thus, it was confirmed that the cross-linking between SPAEK-COOH and CNC can be an efficient membrane candidate for fuel cells [118].

**Figure 7:**
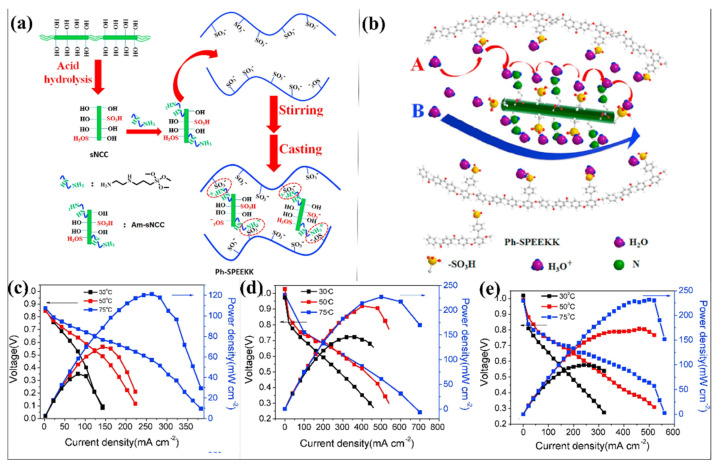
Schematic representation of Ph−SPEEKK and Am−sCNC cross-linked membranes (**a**) preparation process and (**b**) proton transport pathways. (**c**) PEMFC performances at different temperatures (30 °C, 50 °C, and 75 °C): (**c**) Ph−SPEEKK, (**d**) Ph−SPEEKK−Am3−sCNC−5, and (**e**) Ph−SPEEKK−Am3−sCNC-8 membranes. Reprinted with permission from Ref. [121]. Copyright © 2018 Elsevier B.V. (License Number: 5413030856134).

Bano et al. developed sulfonated poly (ether ether ketone) (SPEEK) nanocomposite membranes with CNC and ethylene glycol (EG) [132]. In this case, CNC and EG were used as reinforced and cross-linkers, respectively. Using the solvent casting process, the cross-linked SPEEK-based composite membrane was developed. Without sacrificing conductivity, the cross-linking procedure aids in improving the dimensional stability and strength of the raw SPEEK membrane. By reacting with the ionic moieties of polymer matrices via their SO_3_H and surface OH groups, CNCs considerably increase the strength and offer an efficient channel for proton transport in a membrane. At 95 °C and 95% RH, a notable proton conductivity of 0.186 S/cm was attained for the 4 wt.% loading of CNC cross-linking with SPEEK membrane, equivalent to Nafion 117. In addition to having strong proton conductivity, all created composite membranes had good thermal and oxidative stability [132]. In another study, the high molecular weight of phenylated polymeric precursors was used to create a sulfonated poly(ether ether ketone ketone) (Ph-SPEEKK) [121]. This process has been carried out as a direct sulfonation process in a modest reaction environment. To further improve the performance of Ph-SPEEKK membranes, a modified CNC was introduced in the polymer matrix (Figure 7a). CNCs as aminated and sulfonated CNC (Am-sCNC) were chemically modified in two steps. Using sulfonic acid and 3-trimethoxysilyl propyl ethylenediamine, the CNC was sulfonated and aminated, respectively. Based on the silane coupling agent amount (0.15 mL and 0.45 mL), the amination occurred in Am-sCNC, named Am1-sCNC and Am3-sCNC. Solution casting was used to create the homogeneous and flexible composite membranes made of the functionalized CNC and the Ph-SPEEKK matrix. This composite membrane carries the sulfonic acid groups, silane, and amino properties (Am-sCNC). The water uptake properties at 80 °C of Ph-SPEEKK, sCNC-5, (different ratio of modified CNC in Ph-SPEEKK) Am1-sCNC-2, Am1-sCNC-5, Am1-sCNC-8, Am3-sCNC-2, Am3-sCNC-5, and Am3-sCNC-8 is 46.7 ± 2.3, 49.9 ± 4.6, 48.1 ± 3.7, 54.7 ± 4.7, 52.8 ± 4.3, 58.4 ± 4.6, 58.3 ± 3.9, and 61.6 ± 3.4 %, respectively. The proton conductivities of Ph-SPEEKK, sCNC-5, Am1-sCNC-2, Am1-sCNC-5, Am1-sCNC-8, Am3-sCNC-2, Am3-sCNC-5, and Am3-sCNC-8 is 0.078, 0.102, 0.120, 0.127, 0.107, 0.126, 0.133, and 0.090 S cm^−1^, respectively, at 80 °C. The Ph-SPEEKK- Am3-sCNC-5 provides excellent proton conductivity performance among the other membrane under similar conditions at 80 °C. The proton transfer “Vehicle” and “Grotthus” mechanisms may function more efficiently across the water molecules in the membranes with the presence of free high energy -SO_3_. Similarly, the presence of -SO_3_H, -NH_2_, and -OH functional groups in the Ph-SPEEKK- Am3-sCNC-5 membrane encouraged the transit of proton through the “Vehicle” and “Grotthus” mechanisms (Figure 7b). Moreover, the fuel cell performance (Figure 7c–e) of the Ph-SPEEKK- Am3-sCNC-5 membrane is effectively higher than the Ph-SPEEKK membrane alone, where the obtained power densities are 104 mW cm^−2^ and 227 mW cm^−2^ for Ph-SPEEKK and Ph-SPEEKK- Am3-sCNC-5 membranes, respectively. According to these studies, it can be understood that the cellulose derivatives can effectively influence the performances of PEEK-based polymer membranes [121].

### 3.4. Different Kinds of Polymer Membrane Containing CNC Derivatives

**Figure 8:**
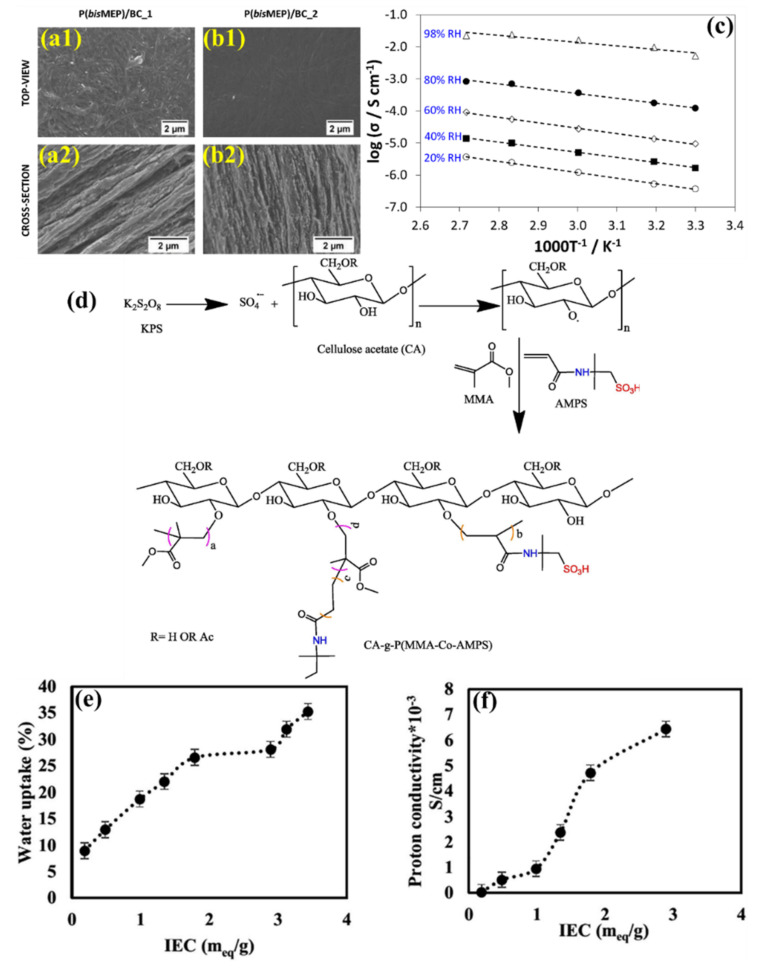
SEM micrograph (**a1**,**b1**) surface and (**a2**,**b2**) cross-sectional view of P(*bis*MEP)–BC nanocomposites membranes. (**c**) Proton conductivity (includes Arrhenius plot) of P(*bis*MEP)–BC membrane at different RH [122]. (**d**) Diagram showing the technique for copolymerizing AMPSA and MMA onto CA. (**e**) Water uptake and (**f**) proton conductivity of CA–g–p(AMPAS–co–MMA) membranes with respect to IEC. Reprinted with permission from Ref. [123]. Copyright © 2021 The Authors. Published by Elsevier B.V. on behalf of King Saud University. (License Number: 5413040027275).

For developing CNC-containing membranes, different kinds of polymers, such as poly(acrylic acid), Poly(*bis* [2-(methacryloyloxy)ethyl] phosphate, poly (2-acrylamido-2-methylpropane sulfonic acid-co-methyl methacrylate), Poly(vinylphosphonic acid)-b-polystyrene, polyvinyl alcohol, polyaniline, and sulfonated Polystyrene have been considered. In this connection, different kinds of cellulose derivatives, such as CNC, CNF, cellulose acetate, and bacterial cellulose, were utilized with different polymers for preparing the PEM for fuel applications. Through flexible mixing and subsequent polymerization, a physically robust and highly conducting poly (acrylic acid) (PAA) electrolyte, including CNFs, was created by Lei Li et al. The PAA electrolyte mechanical strength is increased to 1.875 from 0.656 MPa with a loading of 3 wt.% CNF due to the reasonable hydrogen bonding and physical entanglement. Moreover, half of the initial degree of dimensional swelling is inhibited. Because of the effective ion-transfer routes made possible by the hydroxyl groups revealed upon the surface of CNF in the composite electrolyte, the ionic conductivity is increased by 100% [133]. Vilela et al. applied a newly developed polymer network structure with biocellulose and poly(*bis* [2-(methacryloyloxy)ethyl] phosphate) [P(*bis*MEP)] [122]. Inside the BC three-dimensional network, an in situ free-radical polymerization of *bis*MEP was attained in an environmental reaction process. Based on the in situ free radical polymerization and the weight of the materials in the composite, two kinds of composites were made, P(*bis*MEP)/BC-1 and P(*bis*MEP)/BC-2. The resultant polymeric nanocomposites show high ion exchange capacity, water-uptake ability, mechanical properties and thermal stability. The P(*bis*MEP)/BC composite membranes have high mechanical properties of Young’s modulus >2 GPa and demonstrate thermal stability up to 200 °C. Figure 8a,b represents the surface and cross sectional micrograph images of P(*bis*MEP)/BC-1 and P(*bis*MEP)/BC-2 membranes, respectively. These membrane preserved the lamellar microstructure and three dimensional nano-fibrillar network typical of a BC topology. Among them, Nanocomposite P(*bis*MEP)/BC 1 with a greater BC concentration (79 wt.%) revealed the BC nature. This membrane’s alternating nano-fibril layers of BC and the P(*bis*MEP) phase give rise to a remarkably anisotropic microstructure. In the P(*bis*MEP)/BC-2 membrane, a homogeneous distribution of P(*bis*MEP) was observed inside the BC network, revealed by the confirmation of phosphorus element in the EDX mapping. As mentioned in Table 1, the determined water uptake properties of BC, P(*bis*MEP)/BC-1, and P(*bis*MEP)/BC-2 membranes are 121 ± 11, 79 ± 6, and 155 ± 8%, respectively, at room temperature. The IEC of P(*bis*MEP), P(*bis*MEP)/BC_1 and P(*bis*MEP)/BC_2 are 3.5 ± 0.02, 1.1 ± 0.12 and 3.0 ± 0.05 mmol g^−1^. Figure 8c shows the P(*bis*MEP)/BC-2 membrane proton conductivity (in-plane) at various RH. The proton conductivity of P(*bis*MEP)/BC_1 and P(*bis*MEP)/BC-2 is 22.4 mS cm^−1^ at 94 °C (98% RH) and 27.2 mS cm^−1^ at 80 °C (98% RH), respectively. The key addition is the utilization of a bi-functional monomer which eliminates the requirement for a cross-linker to keep the polyelectrolyte within the BC-network [122]. Developing graft copolymers of cellulose acetate-based membranes showed considerable benefits for fuel cell applications [123]. Through the free-radical polymerization process, cellulose acetate and poly (2–acrylamido–2-methylpropane sulfonic acid–co–methyl methacrylate) have been used to synthesize the copolymer of CA-g-P(AMPS-Co-MMA. As shown in Figure 8d, 2-acryloyamido-2-methylpropane sulfonic acid (AMPS), methyl methacrylate (MMA), potassium persulfate (KPS) initiator, and Cellulose acetate (CA) were used to develop the CA-g-P(AMPS-Co-MMA. The performance of the membranes has been investigated based on grafting. The water uptake and proton conductivity (Figure 8e,f) of the membranes increased by increasing the IEC in the membrane. By forming uninterrupted hydrophilic pathways from the ionic groups already present in the membrane, the ability to absorb water was improved. In the CA-g-P(AMPS-Co-MMA membrane, the number of hydrophilic groups like -CONH and -SO_3_H increased as the AMPS concentration rose. Moreover, there was no doubt about the -SO_3_H group’s role to the IEC’s rise. The proton conductivities are 0.035 × 10^−3^ S/cm and 6.44 × 10^−3^ S/cm for CA and A-g-P(AMPS-Co-MMA membrane, respectively. The physical properties of the membrane may be adjusted with the right graft copolymer composition for efficient fuel cell performance [123].

**Figure 9:**
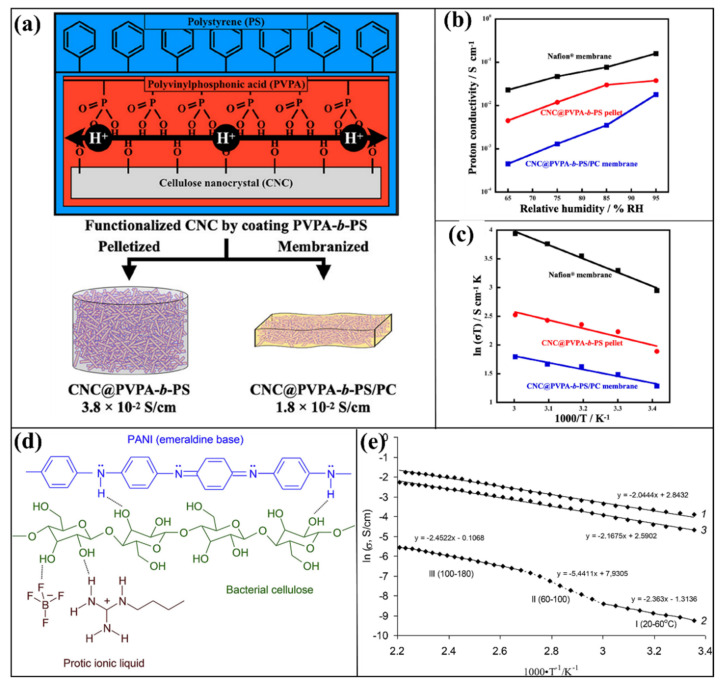
(**a**) Schematic representation of poly(vinylphosphonic acid)–b–polystyrene-coated CNC synthesis processes and mechanism of proton transportation. The CNC@PVPA–b–PS, CNC@PVPA–b–PS/PC, and Nafion membranes (**b**) proton conductivity plots (60 °C, various RH) and (**c**) 95% RH–Arrhenius plots (20–60 °C). Reprinted with permission from Ref. [124]. Copyright © 2022, American Chemical Society. (**d**) Synthesis of BC/PANI-guanidinium-based ionic liquid (BG-BF_4_). (**e**) Ionic conductivity (Arrhenius plots): (1) BG–BF_4_ ionic–liquid, (2) BC/PANI–BG–BF_4_ (80%), and (3) BC/PANI–BG–BF_4_ (95%). Reprinted with permission from Ref. [126]. Copyright © 2018 Elsevier Ltd. (License Number: 5413040320645).

CNC containing a low-acidity polymer electrolyte membrane was developed for fuel cell applications [124]. A polymerization with particles (PwP) technique was utilized to make the core particle function. In this case, using the RAFT polymerization process, novel co-polymers made of hydrophobic polystyrene (PS) and proton-conducting properties of poly(vinylphosphonic acid) (PVPA) were applied to the CNC surfaces (Figure 9a). To develop the membrane, two techniques were used, pelletized and membranized. Moreover, the weak acids behavior of PVPA was selected as a proton conducting agent. Generally, PVPA has a high degree of ionization and ion valence (ion concentration), which can effectively influence the proton conductive behavior. As shown in Figure 9b,c, the proton conductivity of CNC@PVPA-b-PS and CNC@PVPA-b-PS/PC was 3.8 × 10^−2^ and 1.8 × 10^−2^ at 60 °C (95% RH). However, the proton conduction performance of the CNC@PVPA-b-PS and CNC@PVPA-b-PS/PC membranes was lower than the Nafion membrane (1.6 × 10^−1^ at 60 °C (95% RH)), though this sort of filler-filled type membrane has maximum proton conductivity and uses a mild acidity from a phosphonic acid group [124]. Etuk et al. investigated the performances and possibilities of CNC in the form of sulfonation and introduced the desired concentration of 1,2,4-triazole and polyvinyl alcohol (PVA) [125]. To develop the hybrid membrane, a similar (1:1) ratio of sulfonated CNC and PVA was mixed. Afterwards, the desired amount of 1,2,4-triazole (2.0, 3.0, 4.0, and 5.0 mmol) was introduced into the sCNC and PVA combination to develop hybridized membranes. The obtained proton conductivity is 0.0986 (0.0918), 3.1 (2.0), 4.54 (2.82) a 13 mScm^−1^ (8.4 mS cm^−1^) at 120 °C (140 °C) for the 2.0, 3.0, 4.0, and 5.0 mmol of 1,2,4-triazole in the sCNC-PVA membranes, respectively. Based on the proton conductivity measurement results, it can be concluded that the hybrid membrane includes sulfonated CNC and PVA with 1,2,4-triazole can also be a promising membrane candidate for fuel cell applications [125].

For preparing a new kind of membrane, N-Butylguanidinium Tetrafluoroborate (BG-BF4) is a different protic ionic liquid that has been accomplished using a one-step process [126]. At ambient temperature, the ionic liquid exhibits an ionic conductivity of 8 × 10^−3^ S/cm and is thermostable above 300 °C. In this connection, to enhance the performance of bacterial cellulose (BC), it has indeed reacted with BG-BF4 to create novel proton-transporting membranes. At 180 °C, it was exposed that the hybrid BC/BG-BF4 had an ionic conductivity of 4.5 × 10^−4^ S/cm and a tensile strength of 35 MPa. However, the composite membrane BG-BF4 attained lower tensile strength of 6 MPa by increasing the higher loading of BC with a protic ionic liquid weight of 95 percent. But, the high saturation in BC provides 5.2 × 10^−2^ S/cm at 180 °C of good ionic conductivity. To further enhance the performance of BC/BG-BF4, the composite was developed with the further addition of polyaniline (PANI, emeraldine base), as shown in Figure 9d. The conductivity (Figure 9e) of the BC/BG-BF4 (80%) combination was greatly improved to 4 × 10^−3^ S/cm at 180 °C because of the modification of the cellulose nanofibril surface by the oxidative polymerization of aniline. Given that the BC matrix holds ionic liquid very well, it provides considerable benefits regarding proton conductivity, mechanical strength, and thermal stability. This study suggests that these membranes can be used at high temperatures, and in the absence of water, for fuel cell systems [126]. In another report, phosphoric acid containing bio-cellulose with sulfonated polystyrene (sPS) was studied for the fuel cell membranes [127]. Introducing phosphoric acid into the bio-cellulose with sulfonated polystyrene (sPS) membrane provides significant differences in the proton conductivities. The proton conductivity of sPS—bio-cellulose—phosphoric acid obtained in the lower value, where the proton conductivity of Nafion, sPS-Biocellulose, sPS-Biocellulose- phosphoric acid (0.04%) and sPS-Biocellulose- phosphoric acid (0.2%) are 19.04 × 10^−3^, 7.17 × 10^−3^, 2.02 × 10^−3^ and 3.12 × 10^−3^ S/cm, respectively [127].

### 3.5. Cellulose Derivatives Membrane with Inorganic Additives

**Figure 10:**
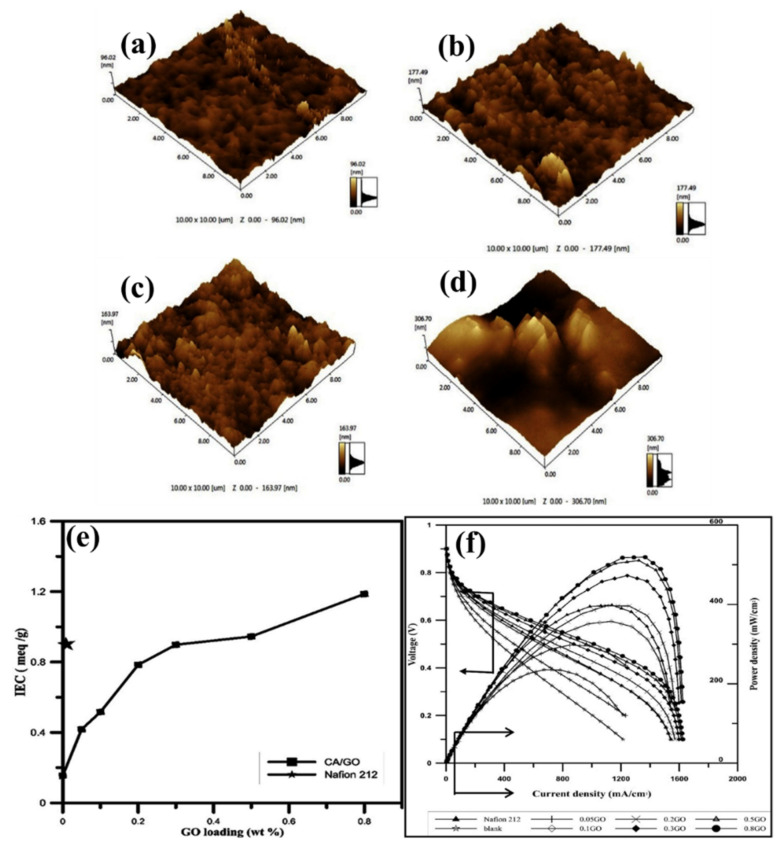
AFM images of cellulose-acetate reinforced graphene oxide nanosheet membrane with different GO content in CA (**a**) GO−0, (**b**) GO−0.1, (**c**) GO−0.3, and (**d**) GO−0.8. (**e**) IEC and (**f**) PEMFC performance of CA-GO membrane concerning the addition of GO with CA. Reprinted with permission from Ref. [128]. Copyright © 2022 The Authors. Published by Elsevier B.V. on behalf of King Saud University. (License Number: 5413040549212).

The developments of hybrid structured polymer electrolyte membranes with the incorporation of inorganic fillers in various polymer matrix provides significant benefits such as increasing oxidative stability, thermal stability, mechanical stability, dimensional stability, and, in certain cases altering the water absorption properties, ion exchange capacity, and ionic conductivity. Graphene oxide (GO) has been considered and served as one of the excellent inorganic polymer fillers to various polymers, namely Nafion, poly(vinyl alcohol), sulfonated poly(arylene ether sulfone), polyvinylidene fluoride, etc., for altering the physicochemical properties [134,135,136,137]. The layered structure of GO primarily contains carboxyl, hydroxyl, and epoxide functional groups. Besides, GO is a connective layer that stands out for its layered composition of inorganic additives, significant aspect ratio, and cost-effectiveness [138]. Madih et al. showed, for the first time, that an effective and extremely durable cellulose acetate (CA)-based membrane containing layered GO is produced using a facile casting procedure [128]. GO’s effective preparation and incorporation into the CA polymer matrix was at different concentrations of GO (0.05, 0.1, 0.2, 0.3, 0.5, and 0.8 wt.%). The surface morphology, height, and phase image of different concentrations of GO in the polymer matrix are shown in the AFM image (Figure 10a–d). As shown in Figure 10a, a smooth and highly homogeneous surface was observed for the CA membrane without GO. Nevertheless, incorporating various amounts of GO filler concentrations in the CA/GO nano-composite membrane showed surface roughness. With the increment of GO filler concentrations (Figure 10b–d) in CA/GO nanocomposite membranes, a higher amount of roughness has been attained. Due to the development of hydrophilic linked channels and the potential existence of foldable nanosheets in the polymer matrices, this rough surface may be explained. Additionally, it has been discovered that including the filler added a little roughness to the nanocomposite membranes’ surfaces without significantly affecting their smoothness and homogeneity. Protons may travel through the membrane more easily because of the increased free volume and water accommodation caused by increased membrane roughness.

The introduction of GO in the CA significantly altered the water uptake properties of CA, where the water uptake % is increasing gradually by increasing the GO content in the CA. The obtained water uptake % is 8.96, 11.17, 11.48, 18.97, 20.77, 22.99, and 24.06 for CA, CA-GO (0.05 wt.%), CA-GO (0.1 wt.%), CA-GO (0.2 wt.%), CA-GO (0.3 wt.%), CA-GO (0.5 wt.%), and CA-GO (0.8 wt.%), respectively. In a similar tendency, the ICE and IC of the CA membrane increased by increasing the GO content. As shown in Figure 10e, the IEC of CA, CA-GO (0.05 wt.%), CA-GO (0.1 wt.%), CA-GO (0.2 wt.%), CA-GO (0.3 wt.%), CA-GO (0.5 wt.%), and CA-GO (0.8 wt.%) are approximately 0.15, 0.4, 0.5, 0.8, 0.913, 0.95, and 1.18 meq/g, respectively. The obtained proton conductivities are 1.21, 1.97, 3.79, 6.92, 9.26, 13.41, and 15.5 mS/cm for CA, CA-GO (0.05 wt %), CA-GO (0.1 wt.%), CA-GO (0.2 wt.%), CA-GO (0.3 wt.%), CA-GO (0.5 wt.%), and CA-GO (0.8 wt %). Interestingly, the IEC and IC of and CA-GO (0.8 wt.%) were considerably higher than the commercial Nafion 212 membrane (IEC: 0.913 meq/g, and IC: 6.94 mS/cm) under similar conditions. Thus, an excellent unit cell performance attained the CA-GO (0.8 wt.%) compared to CA and Nafion 212 membranes at 60 °C and 100% RH. The resulting unit performances are shown in Figure 10f. The obtained maximum power density is 401, 235, and 519 mW/cm^2^ for fuel cells constructed with Nafion 212, CA, and CA-GO (0.8 wt.%), respectively. The incorporation of GO in CA can provide excellent fuel cell performances because of its intrinsic properties, which can significantly alter the physiochemical properties of CA-based membranes [128]. Convincingly, the cellulose structure containing membranes showed improved performances in most composite membranes, as represented in Table 1.

## 4. Developments of Anion Exchange Membranes with Modified Cellulose for Alkaline Fuel Cell

As in PEMFC, the ion exchange membrane is the central part of the AFC device, the most sensible part for conducting ions from cathode to anode, and it properly divides the cathode chamber and anode chamber to avoid short circuiting [139,140,141,142,143]. To properly function the AFC, the desired anion exchange functional properties of the AEM candidate have been used. In an AFC system, AEM’s primary role is transporting the anions (specifically OH^−^) from the cathode side to the anode side during the unit cell operation at various alkaline conditions. The performance of AFC predominantly depends on the function of AEM. Thus, researchers worldwide focus on highly efficient AEM with the following properties: high chemical stability at different alkaline conditions, outstanding anion (OH^−^) exchanges and transport behaviour, cost-effectiveness, lower water drag, and higher mechanical and thermal stability [139,144]. The AFC is commonly interested in quaternary ammonium (−NR_3_^+^) containing membranes. In this case, the quaternary ammonium is firmly bonded to the polymer’s backbone, and the anion is dissociated in the liquid phase. The AFC performance was related to the interaction between the OH^−^ anions (negatively charged) and hydrophilic cation (positively charged) functional groups in the AEM [139]. Thus the developments of a wide variety of quaternary ammonium functionalized polymers, such as poly(arylene ether sulfone), poly(arylene ether ketone), poly (p-phenylene-co-aryl ether ketone), poly(isatin biphenylene), poly(ether ether ketone), and poly(phenylene oxide) are considered for AFC applications [145,146,147,148,149,150]. Recently, several nano- or micro-fillers were tested in composite AEMs to improve the performance of AFCs even more [151,152,153,154,155]. In this connection, the surface-modified different bioinspired materials, specifically quaternized, have been developed to be evaluated in the AFC system with and without additives and other polymers. Interestingly, those kinds of materials provide exceptional performances in the membrane regarding increased ion conductivity and cost-effectiveness. In this viewpoint, cellulose is a natural biopolymer considered effective as an AEM because of its abundance, cost-effectiveness, wealthy hydroxyl groups, high viability to introduce various functional groups, higher mechanical and physical properties, and compatibility with polymers for various energy devices, including fuel cells [156,157,158,159]. A different kind of modification in cellulose structures (like quaternized, sulfonated, and acetylated cellulose) has provided considerable attention for developing new types of membranes for the AFC system.

### 4.1. Quaternized Cellulose Containing AEM Membrane

**Figure 11:**
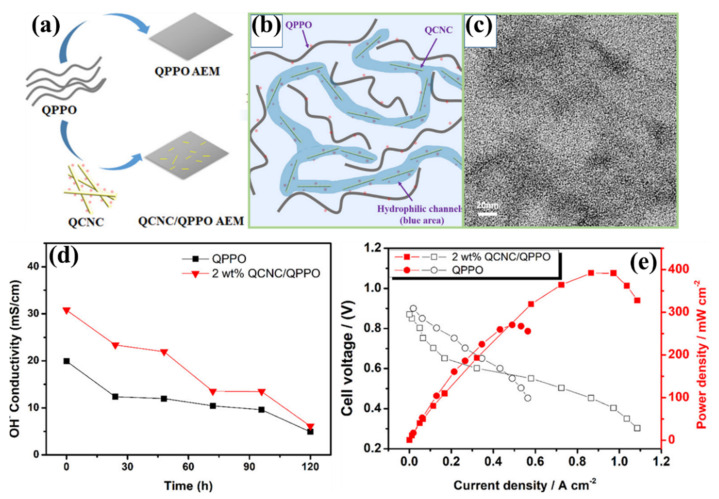
(**a**) Schematic diagram of QCNC–QPPO membrane, (**a**) preparation process, and (**b**) hydrophilic channels in the matrix. (**c**) TEM images of QCNC–QPPO membrane. (**d**) Alkaline stability and (**e**) H_2_/O_2_ fuel cell performance of pure QPPO and QCNC–QPPO membranes. Reprinted with permission from Ref. [175]. Copyright © 2018, American Chemical Society.

Poly(phenylene oxide) (PPO) is one of the polymer’s simple aromatic structures and is considered an excellent polymer candidate material for developing CEM and AEM for various applications [97,160,161,162,163]. Depending on how the membrane needs to work, the ability to change the aryl and benzyl positions in PPO is a beneficial property for making different kinds of membranes [45,97,162,164]. Moreover, PPO possesses a low production cost and efficient hydrolytic, mechanical, thermal, and dimensional stability. So, recently, a lot of attention has been paid to using PPO-based membranes, especially quaternized PPO (QPPO), in AFC systems [165,166,167,168,169,170]. To further improve the physiochemical properties of the QPPO AEM membrane by including various polymeric and inorganic materials with different concepts [166,171,172,173,174]. In this connection, the efficient properties of cellulose have been modified with QPPO. Cheng et al. reported the incorporation of quaternized CNC (QCNC) in the QPPO polymer matrix for the first time [175], as represented in Figure 11a. A different proportion between the QCNC and QPPO has been utilized to enhance the overall AEM properties. The incorporation of 0 to 4 wt.% QCNC in the QPPO polymer matrix is termed QPPO, QPPO/QCNC-0.5, QPPO/QCNC-1, QPPO/QCNC-2, QPPO/QCNC-3, and QPPO/QCNC-4. The presence of QCNC in the AEM membrane significantly altered the physicochemical properties of the QPPO membrane. The obtained IEC for QPPO, QPPO/QCNC-0.5, QPPO/QCNC-1, QPPO/QCNC-2, QPPO/QCNC-3, and QPPO/QCNC-4 is 1.00, 1.01, 1.06, 1.05, 1.00, and 1.04 meq g^−1^, respectively. The IEC is very similar to all QPPO-based membranes, but when QCNC is added to the membrane, higher values can be reached. However, a significant improvement was obtained for the QPPO membrane in the OH^−^ ion conductivity after adding QCNC. The determined ion conductivities (as mentioned in Table 2) are 16.7 ± 0.2, 19.3 ± 0.6, 21.3 ± 0.6, 28.0 ± 0.1, 20.5 ± 0.3, and 13.9 ± 0.7 mS cm^−1^ for QPPO, QPPO/QCNC-0.5, QPPO/QCNC-1, QPPO/QCNC-2, QPPO/QCNC-3, and QPPO/QCNC-4 membranes, respectively. By increasing the QCNC content to 2 wt.% in the QPPO membrane, the OH^−^ ion conductivity increases from 16.7 ± 0.2 to 28.0 ± 0.1 mS cm^−1^. Among the different QCNC loadings, the highest performance of ion conductivity in QPPO/QCNC-2 is primarily attained by the efficient dispersion of QCNC in the QPPO polymer matrix (Figure 11b). As shown in Figure 11c, the homogeneously distributed QCNC provides an excellent hydrophilic channel in the QPPO membrane, which can effectively adsorb the water molecules and promote the OH− ion transportation. In addition, the QPPO/QCNC-2 revealed moderate alkaline stability (as represented in Figure 11d) at 80 °C in 1 mol/L NaOH solution during long-term measurements, where the reasonable ion conductivity is attained after 120 h (5.0 mS cm^−1^). However, QPPO/QCNC-2 showed a more significant unit cell performance than the pure QPPO membrane, as shown in Figure 11e. The attained peak power density of pure QPPO and QPPO/QCNC-2 are 270 mW cm^−2^ and 392 mW cm^−2^ at 60 °C without back pressure, respectively. Comparatively, the best performance of the QPPO/QCNC-2 membrane is majorly attained by excellent ion conductivity, which happened to the QCNC in the polymer matrix [175].

To further improve the performance of cellulose-containing QPPO membranes, a new kind of quaternized cellulose fiber (QCF) and quaternized graphene oxide (QGO) with QPPO was developed in a crosslinking concept by Das et al. [176]. As represented in Table 2, incorporating QCF and QGO fillers in the QPPO matrix considerably altered the ion exchange capacity and transport phenomenon. The developments of different ratio of QPPO:QCF:QGO (100:0:0, 100:1:0, 100:0.5:1, 100:1:1, 100:2:0.5, and 100:3:0.5 provides the IEC of 0.85, 1.12, 2.35, 2.64, 2.09, and 1.82 meq g^−1^, respectively. Among the different compositions, QPPO:QCF:QGO in 100:1:1 provides the highest IEC of 2.64 meq g^−1^. Similarly, the QPPO:QCF:QGO with a ratio of 100:1:1 succeeded the ion conductivity of 114.64 mS cm^−1^ at 25 °C, with the ion conductivity of other ratios of QPPO:QCF:QGO (100:0:0, 100:1:0, 100:0.5:1, 100:2:0.5 and 100:3:0.5 is 20.20, 31.93, 79.71, 60.23, and 68.92 mS cm^−1^, respectively. The fillers’ (QCF and QGO) uniform distribution inside the QPPO polymer matrix, which results in a uniform charge distribution, may be the most likely reason for getting efficient ion conductivity. Moreover, the formation of linked ionic channels is a consequence of improved ionic clustering formation. This behavior possibly improved the overall performance of the QPPO-based membranes [176].

### 4.2. Impact of Sulfonated Cellulose and Cellulose Acetate in AEM Membrane

**Figure 12:**
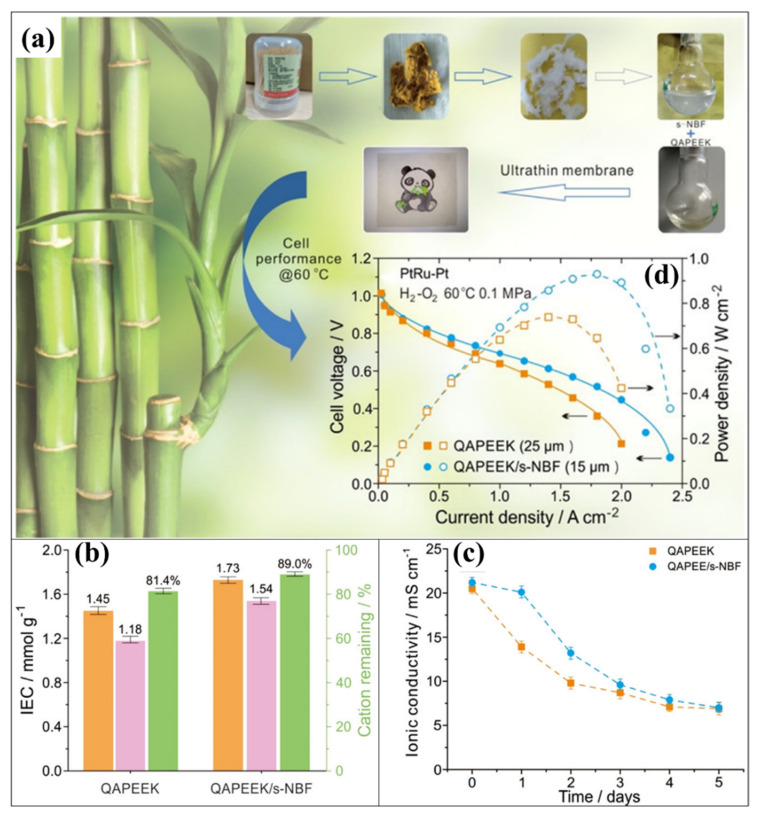
(**a**) Representation of cellulose fiber (CF) derived process from the bamboo and membrane developments QAPEEK membrane with sulfonated CF (SCF). (**b**) IEC, (**c**) IC, and (**d**) fuel cell performances of pure QAPEEK and QAPEEK−SCF membranes. Reprinted with permission from Ref. [177]. Copyright © 2018, American Chemical Society.

Peng et al. developed the cellulose nanofiber from bamboo (CNF—named as Nanobamboo Fiber) for the AFC application [177]. As shown in Figure 12a, the hemicellulose is obtained from the bamboo product (namely the toothpick). Sulfonated CNF (SCNF) was then produced through acid hydrolysis. Here, sulfuric acid was used as a sulfonating agent to make the SCNF. Quaternary-ammonia poly(ether ether ketone) (QAPEEK) has been considered a primary polymer material for developing the composite membrane. An ultra-thin QAPEEK membrane (with a thickness of 15 μm) was designed with SCNF. Despite being extremely conductive, the pure QAPEEK membrane is hampered by excessive water absorption, which causes substantial membrane expansion and poor mechanical stability. To avoid this circumstances without affecting ion conductivity, a wide range of SCNF content has been introduced in the QAPEEK polymer matrix to develop an efficient AEM membrane. Impressively, the swelling degree (SD %) of the QAPEEK membrane is extensively controlled by incorporating SCNF. The attained SD % of different ratios 1:0, 12:1, 8:1, 6:1, and 4.8:1 of QAPEEK:SCNF membrane was 20.0, 17.5, 7.5, 5.0, and 2.5%, respectively. The SD % of the QAPEEK membrane is considerably lowered by increasing the SCNF in the membrane matrix. However, the higher ion conductivity is attuned to the ratio of 12:1 and 8:1 QAPEEK:SCNF membranes than the pure QAPEEK (as mentioned in Table 2), where a lowered ion conductivity observed to higher concentration (for 6:1 and 4.8:1 of QAPEEK:SCNF) of SCNF in QAPEEK. The determined ion conductivities (Figure 12c) are 20.7, 22.0, 21.4, 11.11, and 7.6 mS cm^−1^ at 30 °C for 1:0, 12:1, 8:1, 6:1, and 4.8:1 of QAPEEK:SCNF, respectively. Compared to different ratios, 8:1 of QAPEEK:SCNF membrane provides excellent IEC (1.76 mmol g^−1^), SD (7.5%@30 °C), and ion conductivity (21.4 mS cm^−1^). This 8:1 of QAPEEK:SCNF membrane performance showed better properties than the pure QAPEEK membrane (IEC—1.7 mmol g^−1^, SD—20%@30 °C and ion conductivity—20.7 mS cm^−1^). Accordingly, 8:1of QAPEEK:SCNF is considered an optimum membrane for fuel cell performances, where the attained power density of pure QAPEEK and 8:1—QAPEEK:SCNF were 760 and 930 mW cm^−2^ (Figure 12d), respectively, under similar operating conditions. The dimensional stability and mechanical stability of the QAPEEK:SCNF composite membrane were significantly improved because of the interaction between the sulfonic acid functional group in SCNF and the quaternary ammonia group in QAPEEK [177]. As another option, Samaniego et al. developed a cellulose acetate (CA)-based radiation grafted AEM [178]. The radiation-grafted CA-g-VBC polymer has been prepared with CA film and vinylbenzyl chloride (VBC) monomer using different gamma radiation doses (25, 31, and 40 kGy). To make an efficient anion exchange property in the CA-g-VBC, amine functionalization was carried out using the desired amount of trimethylamine (TMA) for making the TMA functionalized CA-g-VBC. These kinds of membranes reveal efficient ion conductivity (16.3 mS cm^−1^) properties [178]. Based on the studies that have been done, cellulose is a great candidate for a membrane in PEMFC and AFC. Using different ideas, cellulose materials have improved the physicochemical properties and fuel cell performance. The excellent intrinsic properties and commercialization perspectives of cellulose, such as its abundance, facile preparation process, cost-effectiveness, hydrophilic nature, efficient mechanical and thermal properties, renewability, biocompatibility, and intrinsic properties, are the primary reason to get more attention. However, the cellulose’s performance in terms of ion conductivity, mechanical stability, and controlling the gas/methanol permeability must be improved further to make it an excellent membrane for PEMFC and AFC. Moreover, the formation and efficient incorporation of cellulose in the membrane matrix is more challenging where cellulose-based materials may be affected by solvents and other polymers. To overcome these issues, the development of different functional properties of cellulose is required. The ion conductivity of the cellulose materials can be effectively tuned by the functionalization or doping of the cation or anion nature of the functional groups in the cellulose. To avoid lowering mechanical stability after introducing functional groups and enhancing the chemical stability, the crosslinking of cellulose with other polymeric materials in the membrane matrix will be an additional option. Furthermore, there are more options for cellulose-based membranes with various types of organic and inorganic polymer materials. For developing the new kinds of CEM and AEM for hydrogen fuel cells.

## 5. Conclusions

Fuel cell (FC) technologies are developed as a promising alternative to fossil fuels due to their clean emissions, high efficiency, modularity, static nature, wide range of applications, higher hydrogen storage capacity, and fuel flexibility. FC generates electricity directly from the chemical energy of fuel with water and heat as a by-product. Between the different FC ideas, PEMFC and AFC have gotten a lot of attention over the past few decades. In both the FC, ion-exchange membranes (CEM for PEMFC and AEM for AFC) are the vital components for ions transportation between the anode to cathode (in PEMFC) and cathode to anode (in AFC). Due to the high cost of commercial membranes, various efforts have been focussed on biopolymer-based membranes as IEM with high ion conductivity, mechanical, chemical and thermal stabilities. The advantages of cellulose materials, including low-cost, environmental friendliness, renewability, excellent thermal and mechanical stabilities, and biodegradability, provide the opportunity to consider them as an IEM for PEMFC and AFC. This review extensively explains the recent developments (2018 to 2022) of modified cellulose as a membrane candidate or additive for ion-exchange membranes (CEM and AEM). The cellulose structure (CNC and CNF), changes to the cellulose’s natural properties, and the effect of the cellulose structure on different types of synthetic polymers as a CEM or AEM are all explained in detail. Many efforts have been made in recent years to develop cellulose-containing CEM for PEMFC. However, very minimal studies have been reported for AEM with cellulose for AFC. In both types of membranes, the expected structure or ratio of cellulose in the polymer membrane matrix improved the physical and chemical properties, such as the ability to absorb water, the swelling ratio, the ability to exchange ions, and the ability to conduct ions. It increased the stability (thermal, mechanical, and chemical) and fuel cell performance. Based on this review, cellulose and its derivatives can be used as a primary or secondary source of material to make an ion transport hybrid membrane for fuel cell systems with effective properties.

## Figures and Tables

**Table 1:** Physicochemical and unit cell performance results of different kinds of cellulose-containing membranes for PEMFC applications.

Cellulose/Cellulose Derivatives	Polymers/Additives	IEC (meq/g)	Water Uptake (WU) and Swelling Ratio (SR)			Proton Conductivity (IC)		Fuel Cell Test (mW cm^−2^)	Ref.
			T (°C)	WU (%)	SR (%)	T (°C)	IC (S/cm)		
-	Nafion 112	0.91		38		RT	9.0 ± 0.5 × 10^−3^		[119]
						100	9.9 × 10^−2^		[120]
-	Nafion 117	0.92	40	16.5	9	80	95 × 10^−3^	140.8 (at 60 °C and 80% RH)	[46]
CNF	-	0.016	-	-	-	-	-	-	[108]
Sulfonated CNF	-	0.900		26 ± 3	14 ± 5	120	2 × 10^−3^	156 (at 80 °C; 95% RH; 0.1 MPa)	
-	Nafion	0.92	-	26 ± 2	16 ± 2	120	~0.1	-	
Nitro-oxidized carboxy CNF: carboxylate	-	-	-	-	-	80	10.4 × 10^−3^	-	[107]
Nitro-oxidized carboxy CNF: carboxylic acid	-	-	-	-	-	80	14.6 × 10^−3^	19.1 (at 80 °C; 21 psi)	
CMF	-	-	-	-	-	150	1.0 × 10^−8^	-	[109]
CMF	imidazole molecules	-	-	-	-	150	2.7 × 10^−4^	-	
CNC	1H-1,2,3 triazole	-	-	-	-	160	4.0 × 10^−4^ @ anhydrous conditions	-	[110]
CNC	-	-	-	-	-	20 @ ~96% RH	0.4 × 10^−3^	-	[111]
CNC	Sulfosuccinic Acid (SSA)−10%	0.399	-	-	-		4.8 × 10^−3^	-	
CNC	SSA–15%	0.705	-	-	-		7.5 × 10^−3^	-	
CNC	SSA–20%	0.964	-	-	-		11.6 × 10^−3^	-	
CNC	SSA–25%	1.214	-	-	-		12.7 × 10^−3^	-	
CNC	SSA–30%	1.423	-	-	-		14.0 × 10^−3^	-	
CNC	SSA–35%	1.464	-	-	-		10.1 × 10^−3^	-	
CNC—Ramie	-	-	-	21	-	-	-	-	[114]
CNC—Tunicate	-	-	-	21	-	-	-	-	
CNC–Ramie (5%)	Nafion		-	29.5	-	-	-	-	
CNC–Tunicate (5%)	Nafion	1.07 ± 0.01	-	28	-	-	100 × 10^−3^	-	
-	Nafion	1.07 ± 0.01	-	-	-	-	90 × 10^−3^	-	
CNC	-	-	-	-	-	25	0.19 × 10^−4^	-	[115]
Im doped CNC	-	-	-	-	-		1.20 × 10^−4^	-	
Im doped CNC	Nafion (2:1)	-	-	-	-		6.19 × 10^−4^	-	
Im doped CNC	Nafion (4:1)	-	-	-	-		1.09 × 10^−4^	-	
Im doped CNC	Nafion (6:1)	-	-	-	-		4.26 × 10^−4^	-	
-	SFPEEKK-30	0.92	20	7.81	2.11	20	0.024	-	[117]
			90	17.69	7.33	90	0.092	-	
CNC-2%	SFPEEKK-30	0.93	20	13.59	2.81	20	0.029	-	
			90	25.34	11.45	90	0.106	-	
CNC-4%	SFPEEKK-30	0.98	20	16.00	4.16	20	0.030	-	
			90	26.98	14.97	90	0.109	-	
CNC-8%	SFPEEKK-30	0.96	20	11.80	3.30	20	0.028	-	
			90	21.33	12.77	90	0.099	-	
CNC-10%	SFPEEKK-30	0.85	20	9.24	2.39	20	0.013	-	
			90	20.18	9.21	90	0.066	-	
-	SFPEEKK-60	1.72	20	33.95	10.13	20	0.066	-	
			90	92.86	24.43	90	0.152	-	
CNC-2%	SFPEEKK-60	1.91	20	35.06	10.28	20	0.072	-	
			90	104.15	26.34	90	0.189	-	
CNC-4%	SFPEEKK-60	2.03	20	36.29	11.42	20	0.076	-	
			90	115.73	32.6	90	0.245	-	
CNC-8%	SFPEEKK-60	1.98	20	35.08	10.68	20	0.073	-	
			90	107.24	27.43	90	0.210	-	
CNC-10%	SFPEEKK-60	1.84	20	32.58	9.57	20	0.069	-	
			90	79.34	21.27	90	0.172	-	
-	SPAEK-COOH-10	2.013	80	53.95	16.84	80	0.162	-	[118]
-	SPAEK-COOH-30	2.04	80	-	-	80	0.201	-	
CNC-5 composite	SPAEK-COOH-10	2.37	80	58.47	19.65	80	0.177	-	
CNC-8 composite	SPAEK-COOH-10	2.31	80	55.54	18.79	80	0.171	-	
CNC-2 cross-linking	SPAEK-COOH-10	2.15	80	48.95	14.17	80	0.138	-	
CNC-5 cross-linking	SPAEK-COOH-10	2.16	80	50.67	14.20	80	0.152	-	
CNC-8 cross-linking	SPAEK-COOH-10	2.23	80	51.58	14.54	80	0.160	-	
CNC-10 cross-linking	SPAEK-COOH-10	2.19	80	48.02	16.16	80	0.142	-	
-	Ph-SPEEKK	-	80	46.7 ± 2	14.6 ± 1.6	80	0.078	104	[121]
sCNC-5	Ph-SPEEKK	-	80	49.9 ± 4	19.4 ± 1.4	80	0.102	-	
Am1-sCNC-2	Ph-SPEEKK	-	80	48.1 ± 3	11.1 ± 1.9	80	0.120	-	
Am1-sCNC-5	Ph-SPEEKK	-	80	54.7 ± 4	11.9 ± 2.0	80	0.127	-	
Am1-sCNC-8	Ph-SPEEKK	-	80	52.8 ± 4	12.3 ± 2.3	80	0.107	-	
Am3-sCNC-2	Ph-SPEEKK	-	80	58.4 ± 4	11.3 ± 1.0	80	0.126	-	
Am3-sCNC-5	Ph-SPEEKK	-	80	58.3 ± 3	13.8 ± 1.8	80	0.133	227 (at 75 °C)	
Am3-sCNC-8	Ph-SPEEKK	-	80	61.6 ± 3	12.4 ± 1.4	80	0.090	-	
BC	-	–	RT	121 ± 11	-	-	-	-	[122]
-	P(*bis*MEP)	3.5 ± 0.02	-	-	-	-	-	-	
BC_1	P(*bis*MEP)	1.1 ± 0.1		79 ± 6	-	94	22.4 @98% RH	-	
BC_2	P(*bis*MEP)	3.0 ± 0.05		155 ± 8	-	80	27.2 @98% RH	-	
CA					-	-	0.035 × 10^−3^	-	[123]
CA	p(AMPAS-co-MMA)	3.4		27	-	-	6.44 × 10^−3^	-	
-	Nafion	-	-	-	-	60 @ 95% RH	1.6 × 10^−1^	-	[124]
CNC	PVPA-b-PS	-	-	-	-		3.8 × 10^−2^	-	
CNC	PVPA-b-PS/PC	-	-	-	-		1.8 × 10^−2^	-	
CNC:PVA (1:1)	-								[125]
CNC	PVA–2 mmol 1,2,4-triazole	-	-	-	-	120	0.0986 × 10^−3^	-	
		-	-	-	-	140	0.0918 × 10^−3^	-	
CNC	PVA–3 mmol 1,2,4-triazole	-	-	-	-	120	3.1 × 10^−3^	-	
		-	-	-	-	140	2.0 × 10^−3^	-	
CNC	PVA–4 mmol 1,2,4-triazole	-	-	-	-	120	4.54 × 10^−3^	-	
		-	-	-	-	140	2.82 × 10^−3^	-	
CNC	PVA–5 mmol 1,2,4-triazole	-	-	-	-	120	13 × 10^−3^	-	
		-	-	-	-	140	8.4 × 10^−3^	-	
-	BG-BF_4_	-	-	-	-	25	2.1 × 10^−2^	-	[126]
		-	-	-	-	180	1.8 × 10^−1^	-	
BC	BG–BF_4_ (60%)	-	-	-	-	25	3.5 × 10^−5^	-	
		-	-	-	-	180	1.9 × 10^−4^	-	
BC	BG–BF_4_ (80%)	-	-	-	-	25	1.2 × 10^−4^	-	
		-	-	-	-	180	4.5 × 10^−4^	-	
BC	BG–BF_4_ (95%)	-	-	-	-	25	1.6 × 10^−3^	-	
		-	-	-	-	180	5.2 × 10^−2^	-	
BC	PAN—BG-BF_4_ (80%)	-	-	-	-	25	1.5 × 10^−4^	-	
		-	-	-	-	180	4 × 10^−3^	-	
BC	PAN—BG-BF_4_ (95%)	-	-	-	-	25	1.2 × 10^−2^	-	
		-	-	-	-	180	1 × 10^−1^	-	
-	Nafion	-	-	-	-	-	19.04 × 10^−3^	-	[127]
BC	sPS	-	-	-	-	-	7.17 × 10^−3^	-	
BC	sPS+(0.04%) H_3_PO_4_	-	-	-	-	-	2.02 × 10^−3^	-	
BC	sPS + (0.2%) H_3_PO_4_	-	-	-	-	-	3.12 × 10^−3^	-	
-	Nafion 212	0.913	-	-	-	-	6.94 × 10^−3^	401 (at 60 °C)	[128]
CA	-	0.15		8.96	-	-	1.21 × 10^−3^	235 (at 60 °C)	
CA	GO–0.05 wt.%	0.4		11.17	-	-	1.97 × 10^−3^	-	
CA	GO–0.1 wt.%	0.5		11.48	-	-	3.79 × 10^−3^	-	
CA	GO–0.2 wt.%	0.8		18.97	-	-	6.92 × 10^−3^	-	
CA	GO–0.3 wt.%	0.913		20.77	-	-	9.26 × 10^−3^	-	
CA	GO–0.5 wt.%	0.95		22.99	-	-	13.41 × 10^−3^	-	
CA	GO–0.8 wt.%	1.18		24.06	-	-	15.5 × 10^−3^	519 (at 60 °C)	
-	Nafion	1.0	-	-	-	-	-	-	[129]
Cellulose fiber membrane	Nafion	0.15	-	-	-	30	0.007	23 (at 80 °C)	
			-	-	-	90	0.015		
Cellulose fiber membrane	Resorcinol bis(diphenyl phosphate)	0.04	-	-	-	30	0.003	10 (at 60 °C)	
			-	-	-	90	0.010		
Bacterial nanocellulose (BNC)	-	-	RT	21.8 ± 2.1	-	-	-	-	[130]
-	Lignosulfonates (LS)	-		214 ± 5.7	-	-	-	-	
BNC (2:1)	LS	-		55.6 ± 2.4	-	-	-	-	
BNC (4:3)	LS	-		78.0 ± 3.7	-	30	7.3 × 10^−3^	-	
		-		-	-	80	1.9 × 10^−2^	-	
BNC	-	-	RT	22 ± 2	-	94 @ 80 % RH	3.7 × 10^−7^	-	[131]
-	Fucoidan	-		68 ± 3	-		-	-	
BNC—57 wt.%	Fucoidan-43 wt.%	-		45 ± 3	-		1.3 × 10^−4^	-	
BNC—67 wt.%	Fucoidan-33 wt.%	-		32 ± 2	-		7.7 × 10^−5^	-	

**Table 2:** Properties and performance results of modified cellulose-containing Membranes.

Cellulose/Cellulose Derivatives	Polymers/Additives	IEC (meq g^−1^)	Water Uptake (WU) and Swelling Ratio(SR)			Ion Conductivity		Tensile Strength (MPa)	Fuel Cell Test Results (mW cm^−2^)	Ref.
			(°C)	WU (%)	SR (%)	(°C)	(IC) (mS cm^−1^)			
	QPPO	1.00	-	15.1 ± 1.1	2.2 ± 0.1	20	16.7 ± 0.2	28.3 ± 0.9	270 (at 60 °C)	[175]
QCNC-0.5 wt.%	QPPO	1.01	-	18.0 ± 2.2	2.4 ± 0.4	20	19.3 ± 0.6	28.9 ± 2.5	-	
QCNC-1 wt.%	QPPO	1.06	-	17.7 ± 1.4	2.2 ± 0.2	20	21.3 ± 0.6	28.6 ± 0.6	-	
QCNC-2 wt.%	QPPO	1.05	-	16.9 ± 0.4	2.6 ± 0.3	20	28.0 ± 0.1	30.9 ± 0.5	392 (at 60 °C)	
			-	-	-	80	60			
QCNC-3 wt.%	QPPO	1.00	-	16.8 ± 0.2	2.4 ± 0.0	20	20.5 ± 0.3	22.8 ± 2.8	-	
QCNC-4 wt.%	QPPO	1.04	-	17.6 ± 0.8	2.3 ± 0.4	20	13.9 ± 0.7	20.2 ± 2.7	-	
	QPPO	0.85	-	25.34	12.07	RT	20.20	-	-	[176]
QCF-1%	QPPO	1.12	-	40.52	11.56	RT	31.93	-	-	
QCF-0.5%	QPPO−QGO1%	2.35	-	78.87	11.49	RT	79.71	-	-	
						80	157.32	-	-	
QCF-1%	QPPO−QGO1%	2.64	-	88.97	17.21	RT	114.64	-	-	
						80	215.66	-	-	
QCF-2%	QPPO−QGO0.5%	2.09	-	86.19	22.88	RT	60.23	-	-	
QCF-3%	QPPO−QGO0.5%	1.82	-	88.13	25.55	RT	68.92	-	-	
	QAPEEK (1:0)	1.70	30	-	20.0	30	20.7	-	760 (at 60 °C)	[177]
			80	-	27.5	80	110	-		
SCNF	QAPEEK (12:1)	1.66	30	-	17.5	30	22.0	-	-	
			80	-	22.5	80	103	-	-	
SCNF	QAPEEK (8:1)	1.76	30	-	7.5	30	21.4	-	930 (at 60 °C)	
			80	-	7.5	80	119	-		
SCNF	QAPEEK (6:1)	1.59	30	-	5.0	30	11.11	-	-	
			80	-	5.0	80	56.1	-	-	
SCNF	QAPEEK (4.8:1)	1.51	30	-	2.5	30	7.6	-	-	
			80	-	2.5	80	31.5	-	-	
cellulose acetate	vinylbenzyl chloride-grafted (CA-g-VBC)	-	-	-	-	-	16.3	-	-	[178]

## Data Availability

Not applicable.
